# Evaluation of Curcumin Effects on Post-Operative Peritoneal Adhesion in Rats

**Published:** 2012

**Authors:** Vahid Jomezadeh, Amir Hooshang Mohammadpour, Omid Rajabi, Alireza Tavassoli, Ghodratolah Maddah

**Affiliations:** 1 *Department of Surgery, Faculty of Medicine, Mashhad University of Medical Sciences, Mashhad, Iran*; 2 *Endoscopic &Minimally Invasive Surgery Research Centre, Ghaem hospital, Faculty of Medicine, Mashhad University of Medical Sciences, Mashhad, Iran*; 3 *Pharmaceutical Research Centre, Mashhad University of Medical Science, Mashhad, Iran *; 4 *Department of Pharmacodynamics and Toxicology, School of Pharmacy, Mashhad University of Medical Sciences, Mashhad, Iran *

**Keywords:** Curcumin, Post-operative Peritoneal Adhesion, Rat

## Abstract

**Objective(s):**

The purpose of this study holds, for the first time, an evaluation of the intraperitoneal curcumin lavage on the development of post-operative intra-abdominal adhesions.

**Materials and Methods:**

Thirty male Wistar rats were randomized into five groups. The rats were administered anesthesia and underwent surgery in order to create intra-abdominal adhesions. Before the abdomen was closed, five lavage solutions of normal saline (control group), curcumin 1, 3, and 5% and hydrocortisone 1% were used for 1 min. After five days, the rats underwent laparatomy. Based on a histopathology evaluation and serum levels of hs-CRP, TNFα and Isoprostane, peritoneal adhesion severity were compared in different groups.

**Results:**

The groups that received curcumin 3% and 5% showed a significant decrease in TNFα, hs-CRP and Isoprostane serum concentrations compared to the normal saline group, however, these differences were not significant, between the other groups. The intensity of adhesions in the different groups of curcumin 1, 3 and 5% concentrations and hydrocortisone 1% were compared to the normal saline control group and no significant statistical difference was recorded.

**Conclusion:**

Curcumin was not effective in post-operative peritoneal adhesion; however, further studies on curcumin lavage in higher concentrations are recommended.

## Introduction

An adhesion starts to form within 3 hr of surgery and this is a common and unfortunate consequence of most abdominal surgical procedures. Studies have found the incidence of adhesions to be as high as 95% following intra-abdominal surgeries (1). Although advances in surgical techniques such as the use of laparoscopic surgery can help minimize the probability of adhesion formation (2), minimally invasive surgery is not always appropriate or possible. Consequently, intra-abdominal adhesions are still associated with significant morbidity and are considered as a heavy burden on healthcare resources. Estimates of the workload for the treatment of adhesion related disorders in 1994 put the annual cost in the USA at around 1.3 billion US dollars (3). Undoubtedly, these figures will continue to increase because of the growing costs of healthcare and the increase in individuals undergoing surgical intervention due to an aging population (4). Although the mechanism by which adhesions are formed is still poorly understood, a number of steps have now been defined. Abdominal surgery alone causes injury to the peritoneum leading to the activation of the surrounding mesothelium and underlying endothelium, resulting in the localized release of inflammatory cytokines such as tumor necrosis factor alpha (TNF-a) and interleukin-6 (IL-6) into the abdominal cavity. A subsequent recruitment of neutrophils, macrophages and eosinophils and then the release of fibrinous exudates into the peritoneum occur (5, 6). This process is associated with significant oxidative stress from the activation of the mesothelium and underlying endothelial cell and more importantly from the infiltration and subsequent activation of neutrophils and macrophages. Ultimately, this inflammatory process leads to the formation of nascent fibrinous adhesions (7). Whilst many methods have been employed in an attempt to reduce the formation of adhesions, a satisfactory approach to this problem has yet to be found. Although a number of products have been shown to reduce the number and density of adhesions in human studies, an unacceptably high incidence of anastomotic leaks were attributed to the use of these products; hence, limiting the indications of their use. Thus, an effective anti-adhesion formula without similar clinical disadvantages has yet to be bioengineered (8, 9). 

Curcumin the active component in *Curcuma longa* has been seen as a promising solution. It is the main characterized component found in turmeric and is accompanied by desmethoxycurcumin and bis-desmethoxycurcumin derivatives. Curcumin has been shown to be non-toxic and non-mutagenic and exhibits a wide spectrum of biological activities, especially anti-inflammation, antioxidation, anticoagulation, and antifibrotic. The mechanism of the anti-inflammatory effects of curcumin is the ability to inhibit the upregulation of arachidonic acid cascades cyclooxygenase-II (COX-II) and lipoxygenase pathways and the production of IL-8, IL-1β, TNFα, monocyte inflammatory proteins 1 (MIP-1), and monocyte chemotactic protein-1 (MIP-1) (10). Curcumin has been shown to reduce oxidative stress and is a scavenger of hydroxyl, superoxide, and peroxy radicals (11). Based on the pathophysiology of peritoneal adhesion and the anti-inflammatory, antioxidant effect of curcumin, it is hypothesized that curcumin may ameliorate peritoneal adhesions. This study has been the first to investigate the effect of intraperitoneal lavage curcumin on the development of post-operative intra-abdominal adhesions in Wistar rats.

## Materials and Methods

***Chemicals***

In the present study, curcumin (Sigma-Aldrich), F_2_-isoprostane rat serum ELISA Kit (Cayman Co) and Hs-CRP & TNFα rat serum ELISA kits (Bender Med Co) were used. 

***Animals***

Wistar rats used in this experiment weighed 250-300 g and were housed in ventilated rooms at a temperature of 24 ± 2˚C with a 12 hr light/dark cycle and 60 ± 5% humidity. They were freely provided with food and water.

**Table 1:** Comparison of TNFα serum concentration changes in different groups of Wistar rats

	∆ TNFα serum concentration (Pre- Post)*	(*P*-value)**
Normal saline	687 ± 85.75	
Curcumin 1%	555.20 ± 115.14	0.122
Curcumin 3%	298.40 ± 60.94	0.0001
Curcumin 5%	70.4 ± 45.58	0.0001
Hydrocortisone 1%	541.4 ± 86.07	0.074

*Mean±SD

**One-way ANOVA

***Surgical procedure***

The protocols used in our study conformed to guidelines of the Ethical Conduct of Animal Experiments issued by School of Pharmacy and were approved by its ethical committee based on the guidelines of animal experiments in Mashhad University of Medical Sciences. Anesthesia was done using an intraperitoneal injection (IP) of ketamine 100 mg/kg and Xylazin 10 mg/kg. The abdomen was then shaved and prepared with alcohol and an iodine solution. After drying, a 3 cm laparotomy was performed to gain access to the abdominal cavity. In the cecal abrasion group, the cecum was removed and kept moist with saline soaked gauze while dry gauze was used to rub the cecum repeatedly until subserosal bleeding occurred over an area of 1 cm^2^. Time taken for the procedures was kept to a minimum, with the cecal abrasion taking up to 10 min. To simulate an anastomosis, the cecum was incised to create a full thickness abrasion over a length of 1 cm.

The wound was then closed with a continuous 6-0 polypropylene (non-absorbable) suture and the repair leak was tested with a simple pressure test. After intervention, the cecum was then returned to the abdomen and the abdomen wall was closed with a 3-0 polygelatin suture (12).

***Experimental procedure***

Thirty male Wistar rats were divided into five groups of six (control group: normal saline, group I: curcumin 1%, group II: curcumin 3%, group III: curcumin 5%, and group IV: hydrocortisone 1%). Initially, 1 ml of blood from the vein of the tail was taken and the plasma was separated, frozen, and kept at -70˚C. Next, the rats were administered anesthesia and underwent surgery in order to create intra-abdominal adhesions as previously mentioned. Before the abdomen was closed, three lavage solutions of normal saline, curcumin 1, 3, and 5% concentrations and hydrocortisone 1% were applied for 1 min and then the end of the abdomen was sutured and the rats were transferred to the recovery room where they became conscious. They were kept in the recovery room for five days. On the fifth day, a second 1 ml blood sample was taken from the tails and the plasma was separated and frozen at -70˚C. Then, the rats underwent a laparotomy and a cecum and peritoneal sample was sent for a histopathology evaluation and adhesion formation intensity was recorded. 

**Table 2:** Comparison of hS-CRP serum concentration changes in different groups of Wistar rats

	∆hS-CRP serum concentration (Pre- Post)*	)*P*-value)**
Normal saline	3.46 ± 1.51	
Curcumin 1%	5.2 ± 0.74	0.311
Curcumin 3%	6.34 ± 1.3	0.03
Curcumin 5%	8.66 ± 1.035	0.0001
Hydrocortisone 1%	5.92 ± 2.04	0.078

*Mean±SD

**One-way ANOVA

**Table 3:** Comparison of F2-isoprostane serum concentration changes in different groups of Wistar rats

	∆F_2_-isoprostane serum concentration (Pre- Post)*	*P*-value)**)
Normal saline	19.6 ± 6.87	
Curcumin 1%	27.00.± 3.53	0.353
Curcumin 3%	36.8 ±5.8	0.002
Curcumin 5%	58.4 ± 7.23	0.0001
Hydrocortisone 1%	26 ± 6.73	0.406

*Mean±SD

**One-way ANOVA

***Determination of inflammatory and oxidative stress biomarkers***

The blood samples were analyzed according to the relevant ELISA kits. Fs-isoprostane serum concentrations, a reliable index of oxidative stress and TNFα and hs-CRP concentrations, indexes of inflammation, were measured.

***Statistical analysis***

The results were expressed as mean ± standard deviation (SD). Data were analyzed by the one-way ANOVA and χ^2^ test by SPSS 11.5 software. *P*<0.05 was considered significant.

## Results

***Comparison of TNFα serum concentrations***

The changes in TNFα serum concentrations before and after intervention in the groups were statistically compared. The groups that received curcumin 3 and 5% showed a significant reduction in TNFα serum concentration compared to the normal saline group. However, the groups that received curcumin 1% and hydrocortisone did not show any significant reduction of TNFα serum concentration compared to the control group; hence, no significant effect was observed in the curcumin 1% and hydrocortisone groups (Table 1). 

***Comparison of serum hS-CRP***

The changes of hs-CRP serum concentration before and after the intervention of different groups were statistically compared. The groups that received curcumin 3 and 5% had a significant reduction in their concentrations; however, the curcumin 1% and hydrocortisone groups did not show any significant reduction in hs-CRP serum concentration when compared to the control group (Table 2). 

***Comparison of F2-isoprostane serum concentration***

F2-isoprostane serum concentration changes were statistically compared in the different groups before and after intervention and only the group that received curcumin 5% showed a significant reduction in F2-isoprostane serum concentrations compared to the control group. However, this difference was not significant in the other groups (Table 3).

***Comparison of the effect of curcumin on peritoneal adhesions***

The intensity of adhesions in the different groups of curcumin 1, 3, and 5% concentrations and hydrocortisone 1% were compared to the control group and no significant statistical difference was recorded. In the curcumin concentration groups of 3% and 5%, intense adhesion had decreased; however, this decrease was not statistically significant (Table 4).

**Table 4:** Comparison of the effect of curcumin on peritoneal adhesions in different groups of Wistar rats

	Mild adhesion	Moderate adhesion	Severe adhesion	*P*-value*
Normal saline	0	0	5	
Curcumin 1%	0	1	4	1
Curcumin 3%	0	2	3	0.446
Curcumin 5%	0	3	2	0.167
Hydrocortisone1%	0	1	4	1

*One-way ANOVA

## Discussion

Study results indicate that curcumin in 3% and 5% concentrations were able to significantly reduce serum concentrations of TNFα, hS-CRP and Fs-isoprostane when compared to the control group. However, although the 3% and 5% concentrations reduced intense adherence formation, this reduction was not statistically significant.

Curcumin demonstrates various anti-inflammatory and antioxidant properties and these effects have been mentioned in many different clinical and animal studies. Curcumin is found to inhibit upregulation pathways of arachidonic acid cascades cyclooxygenase-II (COX-II) and lipoxygenase by inhibiting the catalytic activities of phospholipase A_2_, Cy1, and D in various cell lines (13). Curcumin inhibits the production of interleukin-8 (IL-8), monocyte inflammatory protein-1(MCP-1), IL-1β, tumor necrosis factor-α (TNFα) from opolysaccharide (LPS)-stimulated monocytes, and macrophages (14). Curcumin also operates through regulating the activities of additional molecular targets in the immune system that control cell adhesion. It has also been shown to be an externally potent inhibitor of the (TNFα) induced expression of intracellular cell adhesion molecule-1(ICAM-1), vascular cell adhesion molecule-1 (VCAM-1) and E-selectin in umbilical vein endothelial cells of rats. Apparently, by inhibiting the induction of steady state transcription levels of ICAM-1, VCAM-1 and E-selectin, curcumin may interfere detrimentally with the TNFα -induced signaling event at an early stage (10, 15).

Several clinical trials have shown that curcumin has an anti-inflammatory effect in rheumatoid arthritis and psoriasis patients (10). Similar to these studies, the results showed curcumin 3 and 5% has a potent anti-inflammatory effect that significantly reduced the serum concentration of TNFα and hs-CRP. The antioxidant property of curcumin extract might be attributed to the presence of chemical groups like hydroxyl, and the 1- and 3-diketone conjugated diene system. Curcumin is able to reduce oxidative stress during inflammatory conditions by down regulating nitric oxide formation, scavenging, and or neutralizing free radicals such as the superoxide anion and H_2_O_2_ known to participate in oxidative chain reactions. Furthermore, the oxidative stress due to acute and sub-chronic inflammation results in the depletion of putative non-enzymatic GSH and enzymatic GPX and SOD antioxidants in target tissues. The depletion of antioxidants observed in different experimental models was significantly restored with curcumin treatment (11, 15). Moreover, the antioxidant activity of curcumin has been demonstrated in different studies and in different disease conditions (16, 17). Similar to these studies, the results of the present study demonstrated that curcumin was a potent antioxidant and that it significantly reduced the serum concentration of F_2_-isoprostane, a reliable indicator of lipid peroxidation.

The study results did not show any significant difference in adhesion intensity between the different groups. In the curcumin 3 and 5% groups, adhesion intensity was reduced when compared with normal saline. In this group, all five rents struck serious adhesion, while in the curcumin 5% group, two rents had serious adhesion, and three rents had intermediate adhesion. This showed the positive effect of curcumin in reducing in-abdomen adhesion after in-abdomen surgery operations, but the reduction was not significant. 

According to the study results regarding the anti-inflammation and antioxidant effects of the curcumin, it seems that its effect depend on the dose. In the two groups receiving curcumin 3 and 5%, TNF serum concentration, hs-CRP and F2 iso-prostan decreased significantly in comparison to the group receiving normal saline. These results are similar to the results of the adhesion intensity as well. However, adhesion intensity decreased in the curcumin 5% group in respect to the group that received normal saline, but the decrease was not significant. Thus, it seems that in order to decrease in-abdomen adhesion significantly, more concentrations of curcumin may be needed.

## Conclusions

It seems that if adhesion induction in the rats was established with less intensity and if a higher concentration of curcumin had been used; we would have had possibly a better and more suitable therapeutic response. Therefore, we suggest that in future researches, more varied adhesion induction methods and higher doses of curcumin be studied. 
